# The role of curcumin against paclitaxel-induced oxidative stress and DNA damage in testes of adult male rats

**DOI:** 10.55730/1300-0144.5556

**Published:** 2022-10-10

**Authors:** Esra BALCIOĞLU, Özge GÖKTEPE, Fazile CANTÜRK TAN, Pınar BİLGİCİ, Birkan YAKAN, Saim ÖZDAMAR

**Affiliations:** 1 Department of Histology and Embryology, Faculty of Medicine, Erciyes University, Kayseri, Turkey; 2 Department of Biophysics, Faculty of Medicine, Erciyes University, Kayseri, Turkey; 3 Department of Histology and Embryology, Faculty of Medicine, Pamukkale University, Denizli, Turkey

**Keywords:** Paclitaxel, curcumin, comet assay, rat, testes

## Abstract

**Background/aim:**

Paclitaxel is a widely used drug for the treatment of cancer, but it possesses toxic effects on male reproductive system. Administering paclitaxel with an antioxidant has become a strategy for preventing the side effects of paclitaxel. Although curcumin is an antioxidant, data concerning the effect of curcumin on paclitaxel-induced testis tissue are lacking. The present study was established to examine the protective impact of curcumin against testicular damage induced by paclitaxel.

**Materials and methods:**

In the study, 40 Wistar albino male rats were used and randomly divided into 4 groups (n:10). The control group received only saline solution; the curcumin group received curcumin throughout the experiment; the paclitaxel group received a total of four doses of paclitaxel on days 1, 7, 14, and 21 of the experiment; curcumin + paclitaxel group received curcumin throughout the experiment and a total of four doses of paclitaxel on days 1, 7, 14, and 21 of the experiment. At the end of the experiment, the rats were decapitated under xylazine and ketamine anesthesia and their testicles were removed. The sections obtained from the testicles were stained with Hematoxylin & Eosin and histopathological damage was evaluated. The TUNEL method was applied to determine apoptotic cells. Testosterone levels were measured in the blood serum. The Johnsen testicular biopsy score (JTBS) was used to evaluate testicular tubules. DNA damage was evaluated in sperm samples taken from the ductus epididymis using the comet assay technique.

**Results:**

Testicular tissue was severely damaged in the paclitaxel group. In the curcumin + paclitaxel group, it was determined that the administration of curcumin with paclitaxel reduced the histological damage in the testicular tissue. Moreover, according to the JTBS, the value was significantly higher in the testicular tubules (p < 0.05). Testosterone levels were higher in curcumin + paclitaxel group than in paclitaxel group. DNA damage also decreased significantly in curcumin + paclitaxel group when compared to paclitaxel group (p < 0.05).

**Conclusion:**

The results showed that curcumin may be protective against damage caused by paclitaxel in the testicles of rats.

## 1. Introduction

Cancer is one of the most important health problems in the world [1]. Chemotherapeutic agents are used in the treatment of various types of cancer, and they are divided into several categories such as alkylates, antimetabolites, mitotic inhibitors, antibiotics, enzymes, hormones and hormone antagonists, and their mechanisms of action are different [2]. However, while chemotherapeutic agents prevent malignant cell division, they cause many side effects such as suppressing the ability of normal marrow cells such as bone marrow hematopoietic cells, testicular epithelium, hair follicles, and oral mucosal epithelium [3,4] and greatly harm patients’ quality of life. Testicular cells have been the target of chemotherapeutic agents due to their ability to easily involve in various miotic and meiotic processes [5].

As a result, spermatogenesis, sperm quality, germ cells, and blood-testis barrier damage were reported in studies [6,7]. Paclitaxel is a member of the taxane class [8] and is an important therapeutic drug used in ovarian cancer, breast cancer, lung cancer, head and neck tumors, and lymphomas [9–11]. However, the use of the drug causes the production of free oxygen radicals (ROS) and DNA damage [12]. Generally, chemotherapy treatment may lead to an increase in oxidative stress and loss of antioxidants. During chemotherapy treatment, antioxidants reduce the severity and frequency of toxicity related to chemotherapy; therefore, it is recommended to use antioxidant substances [13]. Antioxidants act in many ways to eliminate the effects of free oxygen radicals. In addition to preventing lipid peroxidation, they protect protein and nucleic acids and carbohydrates. Moreover, it is known that antioxidants, free radicals, and reaction products increase the activities of biomolecules, phagocytes, and myofibroblasts [14]. Curcumin, known as *Curcuma longa* or turmeric in the literature, is an antioxidant with surprisingly wide beneficial properties due to its complex chemical structure and its ability to affect multiple signal paths [15,16] and it has no known side effects. In addition to being considered an effective antioxidant against oxidative tissue damage, curcumin has anticarcinogenic [17–19], antidiabetic, antiviral, antibacterial, antiinflammatory, antinociceptive, and antimutagenic properties [14,15,20–22]. It has been reported that it is a free radical scavenger [23,24] and a hydrogen provider. Curcumin also inhibits lipid peroxidation [25,26]. It shows its anticarcinogenic and chemical protective effects by effecting transcription factors, growth regulators, adhesion molecules, apoptotic genes, angiogenesis regulators, and cellular signal molecules [14]. In contrast, it has been shown that antioxidant activity is carried out by antioxidant enzymes such as superoxide dismutase, catalases, and glutathione peroxidase [27,28]. ROS can cause DNA damage in the sperm plasma membrane and cause infertility in males with defective spermatozoa [29]. It has been reported that curcumin plays a role in repairing testicular damage due to its antioxidant properties and can be useful in spermatogenic cell preservation and infertility treatment with positive effects on testosterone hormone levels [30].

In recent years, the number of cancer cases is increasing rapidly. Estimated calculations show that one out of four men and five women will be affected by cancer or complications associated with this disease. In order to overcome the negative effects of cancer drugs and investigate their therapeutic effects, combining chemotherapeutics with natural antioxidants can be seen as an ideal approach in clinical studies. Therefore, we aimed to investigate the ameliorative effect of curcumin on paclitaxel-induced testicular damage in terms of apoptosis, histopathological damage, and biochemical and DNA fragmentation.

## 2. Materials and methods

### 2.1. Animals and drug administration

The study protocol was accepted by the Experimental Animal and Local Ethics Committee of Erciyes University (decision no: 15/77). For this study, 40 male adult Wistar Albino rats, weighing 250–300 g, and aged between 8 and 10 weeks, were supplied by the Experimental Animal Laboratory of ERCİYES University. The rats were kept in cages at 21 ± 3 °C and 12 h of light/dark cycle, and given ad libitum access to food and water. After weighing the rats, the animals were randomly divided into 4 groups.

1. The control group: Physiological saline was injected intraperitoneally (i.p.) throughout the experiment.
2. The curcumin group was given 15 mg/kg curcumin [30] (i.p.) throughout the experiment.
3. The paclitaxel group was given a total of four doses of paclitaxel (7 mg/kg) (i.p.) on days 1, 7, 14, and 21 of the experiment.
4. The curcumin + paclitaxel group was given curcumin (15 mg/kg) throughout the experiment and a total of four doses of paclitaxel (7 mg/kg) on days 1, 7, 14, and 21 of the experiment.

The rats were weighed before starting the experiment. Body measurements were repeated with each paclitaxel administration to determine whether curcumin and paclitaxel had an effect on body weight. At the end of the experiment, rats were decapitated after intraperitoneal ketamine (75 mg/kg) + xylazine (10 mg/kg) anesthesia, blood samples and testis tissues were removed. The sections obtained from the testicles were stained with Hematoxylin & Eosin (H&E) and the histological damage was evaluated. Apoptotic cell count was determined by staining with TUNEL method. Testosterone (TES) levels were measured in the blood serum. The Johnsen testicular biopsy score (JTBS) was used to evaluate the testicular tubules. DNA damage was evaluated in the sperm samples taken from the ductus epididymis using the comet assay technique.

### 2.2. Hematoxylin & Eosin staining

Sections of 5 μm taken from the paraffin blocks were spread out on slides. Standard histological methods were applied to the prepared slides. The paraffin was removed with xylol and passed through a graded alcohol series and diluted. The sections were stained with H&E to observe the general histological structure. The slides were then examined under an Olympus BX51 microscope (Tokyo, Japan) (Olympus BX51, Tokyo, Japan).

### 2.3. The Johnsen testicular biopsy score

The Johnsen testicular biopsy score (JTBS) was used to evaluate the degree of damage to the tubules. JTBS use a ten-point scoring system for quantifying spermatogenesis according to the profile of the cells encountered along the seminiferous tubules. A Johnsen score of 10 indicates maximum spermatogenesis activity, whereas a score of 1 indicates complete absence of germ cells [28]. The results of the histological examinations were evaluated by 2 specialist histologists at the Department of Histology and Embryology of the Erciyes University Medical School. Twenty different tubules from 10 different sections randomly selected from each group were examined in 20× magnification. Tubules were evaluated by two specialist histologists and the average JTBS was calculated. The data obtained were processed on a form that had been prepared previously (Table 1).

### 2.4. Biochemical analysis

Biochemical analysis was performed according to the manufacturer’s instructions of the enzyme-linked immunosorbent assay (ELISA) kit. Testosterone levels were determined in all groups using the Enzyme-linked immunosorbent assay (ELISA/CSB-E05100r, 96 Wells kit, CUSABIO Biotech CO., LTD, Wuhan, Hubei, P.R. China) method. The results of the serum samples were given as nmol/mL for the testosterone.

### 2.5. TUNEL assay

A terminal deoxynucleotidyl transferase (TdT)-mediated dUTP nick end labeling (TUNEL) assay was used in order to determine apoptotic cells in all sections of tissues obtained at the end of the experiment. An ApopTag® Fluorescein in situ Apoptosis Detection Kit (EMD Millipore, Darmstadt, Germany) was used for staining. A terminal deoxynucleotidyl transferase (TdT)-mediated dUTP nick end labeling (TUNEL) assay was used in order to determine apoptotic cells in all sections of tissues obtained at the end of the experiment. An ApopTag® Fluorescein in situ Apoptosis Detection Kit (EMD Millipore, Darmstadt, Germany) was used for staining. Sections of 4–5-μm thickness from paraffin blocks were taken into the polysine-coated slides. The sections were exposed to xylol to remove paraffin and passed through graduated alcohol series to take the sections into PBS. After washing with PBS, the sections were incubated for 5 min in equilibration buffer at room temperature. They were then incubated in the TUNEL reaction mixture (TdT enzyme solution+ labelling solution) for 1 h at 37 °C in the dark. The next steps after this stage will be done in the dark. At the same time, washing buffer was prepared with PBS and taken to 37 °C. One hour later, without any washing, the sections were taken to the washing buffer for 10 min. They were then washed with PBS for three times. After mixing blocker solution and antibody diluent solution, the sections were incubated with this mixture for 30 min at room temperature. After being washed three times with PBS, the sections were covered with fluoromount with DAPI. All operations were performed in a humid chamber. The same procedures were conducted on the tissue used as a negative control but without adding TdT. The prepared samples were evaluated using a fluorescence microscope (Olympus BX51, Tokyo, Japan). To assess the number of TUNEL-positive apoptotic cells, at least five different areas were photographed on each tissue using a 40× lens. Apoptotic cell numbers were determined by carefully counting TUNEL positive cells using ImageJ software (ImageJ) at the same magnification (40×). Obtained data were evaluated statistically.

### 2.6. Comet assay technique

Diluted sperm samples extracted from epididymis were centrifuged at 300 × *g* for 10 min at 4 °C. The supernatant was removed and the remaining sperm cells were washed with (Ca2+ and Mg2+ free) PBS. Sperm DNA damage were determined using the single cell gel electrophoresis (comet) assay, which was generally performed at high alkaline conditions. Images of 100 randomly selected nuclei from the sperm sample of each animal were analyzed and sperm with fragmented DNA were counted. Observations were made at a magnification of 400× using an Olympus fluorescent microscope (Olympus, BX51, Japan). The damage was determined from the broken DNA tail that migrated from the sperm head and caused the comet. Cells with tails were evaluated as damaged [31].

### 2.7. Statistical analysis

All statistical analyses were done in SPSS 25.0 program (IBM, USA). Distribution of the data was analyzed by the Kolmogorov–Smirnov and Shapiro–Wilk tests. In variables with normal distribution, the comparisons between the groups were performed using one-way variance analysis (ANOVA) with post hoc Tukey test. In the variables that did not show normal distribution, comparisons between groups were made using Kruskal–Wallis analysis, and if there were differences, multiple comparisons were made using the Mann–Whitney U test. p < 0.05 value was considered statistically significant.

## 3. Results

### 3.1. Body weight

Data of body weight measurements during the experiment are shown in Table 2. The average body weight of the rats in each group was compared just before the chemical application (week 1) and there was no significant difference between the groups. In the 2nd week, the average body weight was 295 ± 25.81 g in the control group, 272.10 ± 40.39 g in the curcumin group, 235 ± 39.93 g in the paclitaxel group, and 278.50 ± 35.90 in the curcumin + paclitaxel group. At the beginning of the 2nd week, when the rates of increase in weight were compared between the control group and the paclitaxel group, and paclitaxel and the curcumin + paclitaxel group, statistically significant differences were found in groups (p < 0.05). When the average body weights are compared at the beginning of the 3rd week, a statistically significant difference was observed between the control group and both the paclitaxel and curcumin + paclitaxel groups (p < 0.05). Body weights were measured just before decapitation (4th week) and when the results were examined, there was no significant difference (p > 0.05) between the control group and the curcumin group. However, there was a statistically significant difference between the control and curcumin groups and paclitaxel and curcumin + paclitaxel groups (p < 0.05).

### 3.2. Testis, epididymis, and seminal vesicle weights

Testis, epididymis and seminal vesicle weights after sacrification are shown in Table 2. Average testicular weights were 1.33 ± 0.32 g in the control group, 1.34 ± 0.09 g in the curcumin group, 1.21 ± 0.13 g in the paclitaxel group, and 1.19 ± 0.07 g in the curcumin + paclitaxel group. As a result of the variance analysis, no significant difference was observed between the control group and the curcumin group (p > 0.05), whereas there was a significant difference between the control and curcumin groups and paclitaxel and curcumin + paclitaxel rate groups (p < 0.05). It was determined that testicular weights and seminal vesicle weights were in parallel. When epididymis weights of control and experimental groups were compared; there was a significant difference between the paclitaxel group and both the control and experimental groups (p < 0.05).

### 3.3. Light microscopic examination

**Figure 1:**
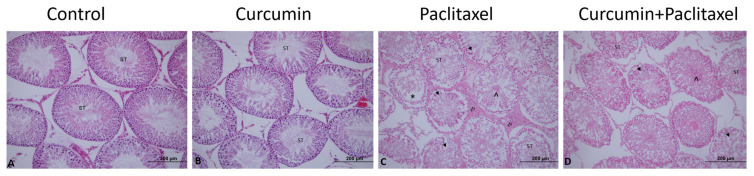
Effects of curcumin on histological changes in the testes caused by paclitaxel. (H&EX20). **A:** Control, **B:** curcumin, **C:** paclitaxel **D:** curcumin + paclitaxel. **ST:** seminiferous tubule, (*): Disorder of seminiferous tubule germinal epithelium, (Arrow): Vacuoles in epithelial cells, **(^):** Desquamation of epithelial cells in the lumen, (➪): Enlarged of connective tissue area.

Testicular tissue from the control group (Figure 1A) and the curcumin (Figure 1B) group showed normal tissue architecture. However, testicular tissue in the paclitaxel group had an irregular tissue architecture compared to the control and curcumin groups (Figure 1C). In contrast to the paclitaxel group, the testicular tissue showed a more regular structure in the curcumin + paclitaxel group (Figure 1D).

**Figure 2:**
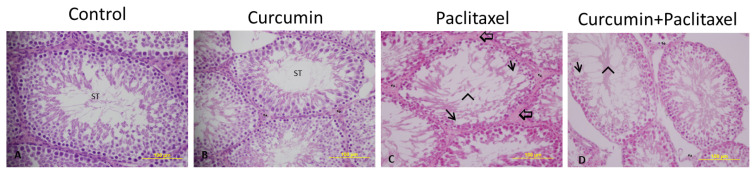
Effects of curcumin on histological changes in the testes caused by paclitaxel. (H&EX40). **A:** Control, **B:** curcumin, **C:** paclitaxel **D:** curcumin + paclitaxel. **ST:** Seminiferous tubule, (Arrow): Vacuoles in epithelial cells, **(^):** Desquamation of epithelial cells in the lumen, (➪): Enlarged of connective tissue area, d⃗↔): Leydig cells.

All four stages of spermatogenesis were observed regularly in the seminiferous tubules of the control (Figure 2A) and curcumin (Figure 2B) groups. Sertoli cells, which contribute to the structure of the blood testicle barrier, also retained their normal structure and location. Leydig cells, responsible for testosterone production in the interstitial space, were also observed in their normal structure. Rarely, separation sites were found on the wall of the seminiferous tubules in to the curcumin group. There was vacuole formation in germinal epithelium and degenerative changes in spermatogenic cells, necrosis and desquamation in some tubules in the testicular tissues of rats that were administered only paclitaxel. Marginal irregularities and damaged epithelium of the paclitaxel seminiferous tubules were observed. In the seminiferous tubule lumen, primary and secondary spermatocytes, spermatids and spermatozoa could not be differentiated in the light microscope. Malformed Sertoli cells appeared separated from the basal laminae and very close to each other. Occasionally, there was thinning in germ cell thickness, as well as corrugation in the basement membrane, and these findings were also compatible with JTBS. The entry of paclitaxel into the testis originates from the connective tissue in the interstitial area. Leydig cells are located between the interstitial connective tissue between the seminiferous tubules and show eosinophilic staining because they secrete androgens. In the testicular tissues of the paclitaxel group, pale eosinophilic staining and indistinct nuclei were observed in Leydig cells (Figure 2C). Damage to Leydig cells was also associated with a decrease in testosterone levels, which was seen in biochemical analysis. In the seminiferous epithelium of control group, Sertoli cells were normally located in the basal compartment and showed normal-shaped nucleus with typical nucleolus; however, in the seminiferous epithelium of the curcumin + paclitaxel (Figure 2D) group, Sertoli cells nuclei with irregular outline, and intense baso-philia were found. Almost no edematous area was found in the interstitial region. Additionally, there were morphological signs of improvement in Leydig cells. These cells had prominent nuclei and dense eosinophilic cytoplasm. Spermatogenic serial cells were significantly close to normal.

### 3.4. JTBS results

JTBS of the control and experimental groups are shown in Table 3. As a result of the analysis of variance, no significant difference was observed between the control group and the curcumin group (p > 0.05), while there was a significant difference between the control and curcumin groups and the paclitaxel and curcumin + paclitaxel groups (p < 0.05). At the same time, it was determined that there was an increase in JTBS of the curcumin + paclitaxel group and this increase was found to be statistically significant (p < 0.05). However, when this increase in JTBS of the curcumin + paclitaxel group was compared to the control group, no significant difference was observed (p > 0.05).

### 3.5. Biochemical results

Plasma testosterone levels of control and experimental groups are shown in Table 3. The mean testosterone level of the control group was 27.47 ± 3.22 pg/mL, the average testosterone level of the group exposed only to curcumin for 4 weeks was 21.20 ± 5.54 pg/mL, the mean testosterone of the paclitaxel group applied once a week for 4 weeks was 17.73 ± 2.36 pg/mL and the testosterone level was determined as 18.42 ± 5.99 pg/mL in the group that paclitaxel and curcumin were applied together. According to these results, there was a decrease in the average testosterone level in all experimental groups compared to the control group and this decrease was found to be statistically significant (p < 0.05). It was determined that testosterone levels decreased due to the damage in Leydig cells in the paclitaxel-treated groups and curcumin applied as an antioxidant, increased testosterone level in the curcumin + paclitaxel group compared to the paclitaxel group, but this increase was not significant compared to the control group (p > 0.05).

### 3.6. TUNEL evaluation

TUNEL staining was performed to determine the apoptotic cells in the testicular tissue. The apoptotic index results are shown in Table 3.

**Figure 3:**
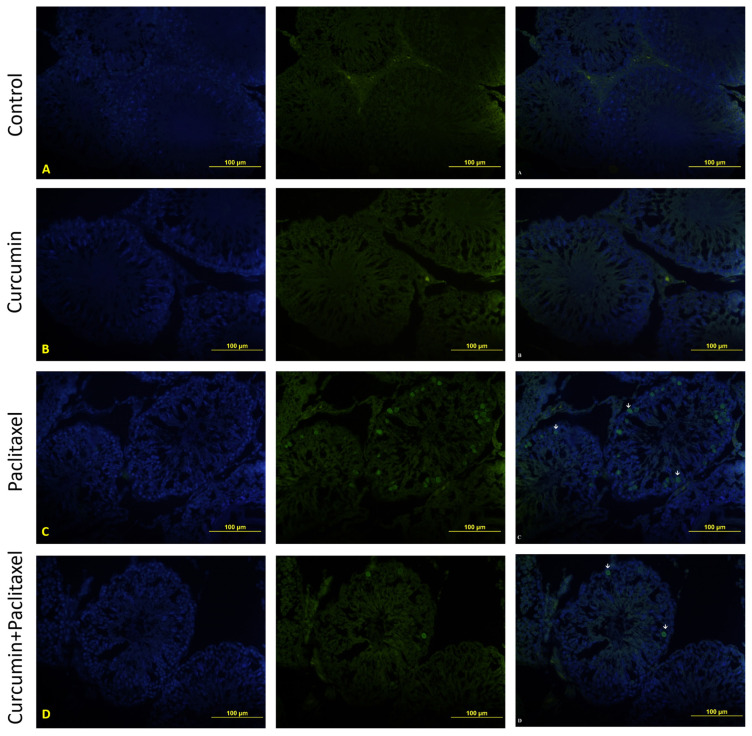
Representative images of TUNEL assay on seminiferous tubule cross-sections from four animals groups. (X40). **A:** Control, **B:** curcumin, **C:** paclitaxel **D:** curcumin + paclitaxel. **(Arrow):** Apoptotic cell.

In the control (Figure 3A), curcumin (Figure 3B), paclitaxel (Figure 3C), and curcumin + paclitaxel (Figure 3D) groups, apoptotic cells were generally determined to be in the spermatogenetic cell line. As a result, the number of apoptotic cells in the seminiferous tubules increased in the paclitaxel group, and this increase produced a significant difference between the paclitaxel group and the control group (p < 0.05). When the number of apoptotic cells in the curcumin + paclitaxel group was compared to the paclitaxel group, there was a significant decrease in the curcumin + paclitaxel group (p <0.05). However, due to the antioxidant application in the curcumin + paclitaxel group, there was a slight decrease in the number of apoptotic cells, although not as much as the control and curcumin groups, but there was a significant difference between the groups (p < 0.05).

### 3.7. DNA damage

The microphotographic appearance of the fragmented DNA sperm and the head and tail DNA numbers in all of the groups are presented in Table 4, respectively. According to the data obtained as a result of measuring the microphotographic images, decreasing the amount of head DNA or increasing the amount of tail DNA resulted in DNA fragmentation in the cell. Only in the paclitaxel administration group, the sperm DNA fragmentation rate significantly increased when compared to the control group (p < 0.05).

**Figure 4:**
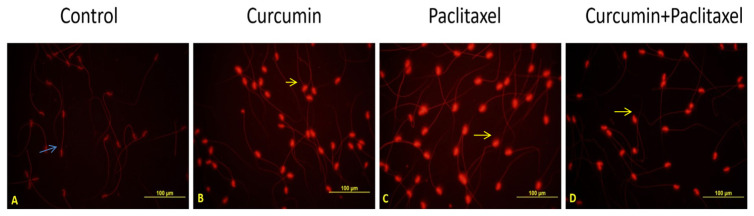
Sperm cells processed using single cell gel electrophoresis (Comet) assay. **(A)** Intact cells with undamaged DNA (without comet tail) in control group. **(B)** Cells with minor DNA damage (short Comet tails) in curcumin group. **(C)** Cells with major DNA damage (long Comet tails) in paclitaxel group. **(D)** Cells with moderate DNA damage (considerable heterogeneity of Comet tail lengths within an individual) in curcumin + paclitaxel group. Cells without comet tails were indicated undamaged cells (blue arrows). Cells with short comet tails and cells with long comet tails were indicated damaged cells (yellow arrows) (Ethidium bromide staining, 400×).

The comet assay results of the control (Figure 4A) and curcumin (Figure 4B) groups are shown respectively. The sperm DNA fragmentation rate decreased significantly (p < 0.05) in the curcumin + paclitaxel group when compared to the paclitaxel group. The comet assay results of the paclitaxel (Figure 4C) and curcumin + paclitaxel (Figure 4D) groups are shown respectively. Although the curcumin application alone increased the rate of sperm DNA fragmentation when compared to the control group, this increase was less than that of the paclitaxel group (p < 0.05).

### 3.8. Discussion

Due to the rapid growth and proliferation of cancerous cells, most chemotherapeutic drugs have been developed to destroy cells with such characteristics. However, since it has the ability to divide rapidly, side effects occur in blood cells directly affected by chemotherapy, cells in the gastrointestinal system, hair follicles, and sperm cells. In addition, some chemotherapeutics may also have negative effects on vital organs such as the heart, kidneys, bladder, lungs, and nervous system in general [32,33].

Especially male infertility and sterility is one of the negative factors caused by chemotherapeutic drugs in the reproductive system [33,34]. Adverse conditions such as errors in spermatogenesis, decreases in sperm quality parameters, dysfunction in the ejaculation, and dysfunction in the hypothalamus-pituitary-adrenal axis are among the side effects that chemotherapeutics cause in the reproductive system [32,33,35,36]. While Leydig and Sertoli cells in the testicle are partially resistant to chemotherapeutics, the germinal epithelium is highly sensitive to these drugs [37]. However, depending on the dose and duration of chemotherapy used, it has been shown that spermatogenesis may return to normal within a certain period of time after termination of treatment if the stem cells in the germinal epithelium remain intact [34,38]. In this study, the damage caused by paclitaxel application was determined to be especially in the germinal epithelium. This result is in agreement with both JTBS results and TUNEL staining results. In contrast to the above studies, damage was observed in the Leydig cells in this study. Damage to Leydig cells was also associated with the decrease in testosterone levels achieved by biochemical results. It was concluded that this damage might be due to paclitaxel entering into testicular tissue from the interstitial area.

In a study, it was reported that paclitaxel administration caused a decrease in prostate gland, epididymis and testicular weights, sperm density and motility. However, decrease in spermatogenic cell density and irregularities in Sertoli cells were observed [39]. In the current study, treatment with paclitaxel decreased testis, epididymis, and seminal vesicle weights, necrosis in seminiferous tubules and areas of edema in connective tissue between tubules were observed. Morphological changes were observed in Sertoli cells and spermatogenic series. These cells had vacuolization with an indistinct nuclear structure. Although the effect of oxidative stress induced on male infertility with toxic agents is known, its mechanism has not been fully clarified yet [40]. In a study, it was reported that testicular weight and testosterone level decreased after fludarabine administration in patients with chronic lymphocytic leukemia. It has also been reported that sperm DNA damage, FSH [39] and LH levels are increased [41]. Similarly, it has been found that gemcitabine combined with taxanes caused significant gonadal lesions such as decrease in bilateral testicular weight and inhibin B level and increase in FSH level [42]. In studies performed with methotrexate, it was reported that testicular weight, seminiferous tubule diameter and germinal epithelial thickness decreased and [43] degenerations were formed [44]. In addition, it has been found to have lethal effects on spermatogenic cells, spermatocytes and spermatids [45], reducing the size of Sertoli and Leydig cells [46].

It has also been stated that chemotherapeutics cause DNA damage, apoptosis, decrease in sperm quality and infertility in spermatogenic cells by causing oxidative stress in the male reproductive system with lipid, cholesterol, and protein peroxidation [37,40]. The comet assay method is an important method used to determine the level of damage to DNA [47]. Sarıözkan et al. reported that sperm DNA integrity was induced by Taxans, as observed via the comet assay technique. In their study, the comet assay technique was used to demonstrate that Docetaxel, paclitaxel, and Docetaxel + paclitaxel impaired sperm DNA fragmentation when compared to their control group [31]. In the current study, paclitaxel-induced DNA damage was observed in sperm using the comet assay technique.

These days, complementary and alternative medicine is widely used by both healthy and sick people. With the help of antioxidant substances of different structure and properties, ROS levels are reduced, antioxidant activities are increased, and possible side effects of chemotherapeutics in male reproductive system are tried to be reduced or eliminated. Due to its anticancer and chemoprotective properties, vitamins, chemicals and herbal products are among the most widely used alternative medicine methods by cancer patients [48].

Curcumin is an effective antioxidant that destroys nitric oxide, hydrogen peroxide, and superoxide radicals released from activated macrophages. Its antioxidant property is due to its phenolic structure. It has been reported in the literature that curcumin, which has attracted great attention in recent years, protects spermatogenesis and steroidogenesis against various toxic agents by ensuring the continuity of antioxidant enzyme systems and reducing oxidative stress and lipid peroxidation [40,50].

In the study conducted by Ilbey et al. [16], it was reported that curcumin used as an antioxidant preserves the testicular weight and spermatogenesis against the toxic effects caused by cisplatin, morphological features of the cisplatin + curcumin group are similar to the control group, and no perivascular fibrosis were observed in the interstitial connective tissue. It was also stated that germ cell layer number, seminiferous tubule diameter, and testosterone levels improved in the cisplatin+ curcumin group compared to the cisplatin group. In a 2-month study conducted by Salama et al. [30], curcumin given at a daily dose of 15 mg/kg to rats was shown to increase sperm motility and number and to decrease sperm anomalies.

In the current study, paclitaxel was used to induce oxidative stress-based cytotoxicity in adult rats. Curcumin, which is known to be a natural antioxidant source, was preferred instead of synthetic antioxidants to suppress oxidative stress. When the data were evaluated, it was determined that there was a statistically significant difference between the paclitaxel group and the control group in the testicular, epididymis, and seminal vesicle weight. Improvement in the seminiferous tubules of the testicular tissues of rats treated with curcumin together with paclitaxel was observed. The spermatogenic series cells were very close to normal and there was a decrease in the number of apoptotic cells. The JTBS results showed that the application of curcumin together with paclitaxel reduced the damage caused by paclitaxel. Comet assay findings showed that DNA damage of paclitaxel in spermatozoa was reduced by administration of curcumin. It was determined that blood testosterone level increased in the curcumin + paclitaxel group compared to the paclitaxel group, but this increase was not statistically significant.

As a result, it was determined that curcumin, which has antioxidant properties against paclitaxel-induced testicular damage, contributes to strengthening the cell’s survival mechanism by increasing the internal resources of the cell. It is believed that different and higher doses of curcumin could further reduce the damage.

## Figures and Tables

**Table 1:** Johnsen testicular biopsy score.

Score	Histological findings	Score	Histological findings
**1**	No cells in the tubular section	**6**	There were few spermatids (5/tubule)
**2**	There were only Sertoli cells	**7**	There were too many spermatids without any sign of difference
**3**	Germ cells were just as spermatogonium	**8**	Late spermatids without mature spermatozoa
**4**	There were few spermatocytes (5/tubule)	**9**	There were few spermatozoa (5/tubule)
**5**	There were too many spermatocytes	**10**	Exact spermatogenesis was present with a large number of spermatozoa

**Table 2:** Average weight measurements for control and experimental groups.

Average weight measures					
	Control	Curcumin	Paclitaxel	Curcumin + paclitaxel	p
**1st-week body weight**	297.40 ± 29.43^a^	300 ± 38.49^a^	295.80 ± 30.79^a^	315.70 ± 26.11^a^	>0.05
**2nd-week body weight**	295 ± 25.81^a^	272.10 ± 40.39^ab^	235 ± 39.93^b^	278.50 ± 35.90^a^	<0.05
**3rd-week body weight**	306 ± 36.95^a^	274.50 ± 45.12^ab^	260.20 ± 28.98^b^	248 ± 23.35^b^	<0.05
**4th-week body weight**	299 ± 33.48^a^	287 ± 44.23^a^	230 ± 31.26^b^	231.50 ± 23.81^b^	<0.05
**Testicular weights**	1.33 ± 0.32^a^	1.34 ± 0.09^a^	1.21 ± 0.13^b^	1.19 ± 0.07^b^	<0.05
**Epididymis weight**	0.67 ± 0.10^a^	0.64 ± 0.07^a^	0.52 ± 0.06^b^	0.63 ± 0.05^a^	<0.05
**Seminal vesicle weight**	0.68 ± 0.15^a^	0.64 ± 0.08^ab^	0.54 ± 0.09^b^	0.61 ± 0.15^ab^	<0.05

The same letters on the same line show similarity between groups. Different letters indicate differences between groups.

**Table 3:** Testosterone level. Johnsen testicular biopsy score (JTBS) and apoptotic cell count results.

	Control	Curcumin	Paclitaxel	Curcumin + paclitaxel	p
**Testosterone level**	27.47 ± 3.22^a^	21.20 ± 5.54^b^	17.73 ± 2.36^b^	18.42 ± 5.99^b^	<0.05
**JTB**	7.97 ± 1.41^a^	7.98 ± 1.50^ac^	5.35 ± 1.46^b^	6.91 ± 1.58^d^	<0.05
**Apoptotic cell count**	0.33 ± 0.61^a^	0.48 ± 1.52^a^	3.21 ± 5.80^b^	2.92 ± 4.13^c^	<0.05

The same letters on the same line show similarity between groups. Different letters indicate differences between groups.

**Table 4:** Comet assay results.

Comet parameters (n=300)	Control	Curcumin	Paclitaxel	Curcumin + paclitaxel	p
**L Head (μm)**	85.31 ± 22.00^a^	67.77 ± 10.97^c^	60.46 ± 12.90^b^	62.23 ± 12.42 ^b^	<0.05
**L Tail (μm)**	6.18 ± 5.06^a^	27.37 ± 7.18^c^	39.54 ± 13.52^b^	31.22 ± 9.53^d^	<0.05
**L Comet (μm)**	91.1 ± 23.55^a^	95.11 ± 15.13^a^	102 ± 22.54^b^	91.44 ± 18.37^a^	<0.05
**Head DNA (%)**	98.98 ± 0.58^a^	86.65 ± 2.57^c^	77.75 ± 6.15^b^	85.48 ± 2.79^d^	<0.05
**Tail DNA (%)**	1.01 ± 0.58^a^	13.33 ± 2.56 ^c^	22.34 ± 6.06 ^b^	14.51 ± 2.79 ^d^	<0.05
**TM**	0.03 ± 0.07^a^	3.86 ± 1.43^c^	9.04 ± 5.00^b^	4.66 ± 1.68^d^	<0.05
**OTM**	0.13 ± 0.25^a^	5.28 ± 1.53^c^	7.57 ± 3.45^b^	5.49 ± 1.64^c^	<0.05

The same letters on the same line show similarity between groups. Different letters indicate differences between groups.

* **L Head;** Length head, **L Tail;** Length tail, **L comet;** Length comet, **TM;** Tail moment, **OTM;** Olive tail moment.
